# TH1L involvement in colorectal cancer pathogenesis by regulation of CCL20 through the NF‐κB signalling pathway

**DOI:** 10.1111/jcmm.18391

**Published:** 2024-05-29

**Authors:** Shaochang Wang, Yujing Sun, Chunya Li, Yueyang Chong, Meihong Ai, Yanxia Wang, Haiyun Shi, Yu Shang

**Affiliations:** ^1^ The Key Laboratory of Cell Proliferation and Regulation Biology, Ministry of Education, College of Life Sciences Beijing Normal University Beijing China; ^2^ Department of Laboratory Medicine Peking University International Hospital Beijing China; ^3^ Department of Cancer Precision Medicine, The MED‐X Institute The First Affiliated Hospital of Xi‘an Jiaotong University Xi‘an China; ^4^ Department of Gastroenterology Beijing Friendship Hospital, National Clinical Research Center for Digestive Diseases, Beijing Digestive Disease Center, Beijing Key Laboratory for Precancerous Lesion of Digestive Diseases, Capital Medical University Beijing China

**Keywords:** CCL20, colorectal cancer (CRC), NF‐κB signalling pathway, TH1L

## Abstract

TH1L (also known as NELF‐C/D) is a member of the Negative Elongation Factor (NELF) complex, which is a metazoan‐specific factor that regulates RNA Polymerase II (RNAPII) pausing and transcription elongation. However, the function and molecular mechanisms of TH1L in cancer progression are still largely unknown. In this study, we found that TH1L was highly expressed in colorectal cancer (CRC) tissues and the faeces of CRC patients. Overexpression of TH1L significantly enhanced the proliferation and migration of CRC cells, while its knockdown markedly suppressed these processes. In mechanism, RNA sequencing revealed that CCL20 was upregulated in TH1L‐overexpressed CRC cells, leading to activation of the NF‐κB signalling pathway. Rescue assays showed that knockdown of CCL20 could impair the tumour‐promoting effects of THIL in CRC cells. Taken together, these results suggest that TH1L may play a vital role via the CCL20/NF‐κB signalling pathway in CRC proliferation and migration and may serve as a potential target for diagnosis and therapy of CRC.

## 1 INTRODUCTION

Colorectal cancer (CRC) is the third most common type of cancers and the second leading cause of cancer‐related mortality worldwide.^1^, ^2^ Its incidence is surging in many cities and countries around the world.^3^ By 2030, the global burden of CRC will increase by 60% to 2.2 million new cases, leading to 1.1 million deaths.^4^ Therefore, the demand for more efficient diagnostic and treatment strategies is imperative. The process of CRC tumorigenesis and progression is intricate and driven by multiple genetic and epigenetic alterations, including the activation of oncogenes and the inactivation of tumour suppressor genes that govern cell proliferation, migration, invasion and chemoresistance.^5^ Thus, further research is urgently required to explore malignant factors and analyse the mechanism of CRC progression and metastasis, which could provide potential biomarkers and novel therapeutic targets for CRC patients.

The human trihydrophobin 1‐like (*Th1l*) was initially identified in the analysis of the nearest transcription units 3′ to GNAS1,^6^ which is the homologue of Drosophila *Th1*.^7^ This is also in accordance with the gene‐coding of NELF‐C/D, a component of the human negative transcription elongation factor (NELF) complex.^8^ The NELF complex and the DSIF (DRB sensitivity‐inducing factor) complex work together to restrain transcriptional elongation by RNA polymerase II. Intracellularly, TH1L has been reported to act as a negative regulator of A‐Raf and PAK1, suppressing the MEK/ERK signalling pathway to regulate cancer progression through its impacts on cellular migration, invasion and epithelial‐mesenchymal transition (EMT).^9^, ^10^ Moreover, TH1L can degrade androgen receptor, preventing androgen receptor transactivation and is essential for testis development.^11^ Nevertheless, research on the role and regulation of TH1L in cancer progression is still limited. Previous studies have revealed that TH1L inhibits the proliferation and invasion of cultured breast cancer cells.^12^ Additionally, it has been reported that knockdown of TH1L enhances the sensitivity of gastric cancer cells to epirubicin or cisplatin.^13^ Recently, it was found that knockdown of TH1L suppresses the proliferation of colorectal cancer cells, yet the mechanism remains unknown.^14^

CCL20, a critically important chemokine, belongs to the subfamily of small cytokine CC. In CRC, CCL20 has been found to be dysregulated, with elevated levels observed in tumour tissues and associated with cancer progression and poor clinical outcomes.^15^ One of the key mechanisms by which CCL20 exerts its effects is activating the Nuclear Factor‐Kappa B (NF‐κB) signalling pathway.^16^ NF‐κB is a transcription factor that plays a critical role in regulating the expression of genes involved in inflammation, immune response and cell survival.^17^ Stimulation of NF‐κB signalling in CRC has been linked to tumour growth, resistance to apoptosis and enhanced metastatic potential.^18^ Further investigation of the molecular mechanisms underlying the involvement of CCL20/NF‐κB in CRC may lead to the development of novel therapeutic targets.

In this study, we demonstrated that TH1L is highly expressed in both CRC tissues and faeces of CRC patients. Moreover, by utilizing overexpression and knockdown approaches, we found that TH1L plays a critical role in CRC cell proliferation and migration. Interestingly, we revealed CCL20 as a novel putative target of TH1L. Specifically, the upregulation of CCL20 activates the NF‐κB signalling pathway to promote the proliferation and migration of CRC cells. Our study not only provides new insights into the regulation of CRC progression but also uncovers a new mechanism through which TH1L regulates the CCL20/NF‐κB signalling pathway. Consequently, TH1L may be a new potential target for the molecular diagnosis and treatment of CRC.

## 2 MATERIALS AND METHODS

### 2.1 Patients and specimens

A total of 303 participants treated at Beijing Friendship Hospital, Capital Medical University, were enrolled in this study. The cohort comprised 150 patients diagnosed with CRC, 79 patients with inflammatory bowel disease (IBD) and 74 healthy controls. Patients with any other types of tumours in their past history who received preoperative chemotherapy or radiotherapy were excluded. Informed consent was obtained from all participants in the study. The pathologic information of CRC patients and the demographic data of CRC, IBD and healthy participants are shown in Table 1. This study was approved by the Beijing Friendship Hospital Ethics Committee. Stool samples were collected from patients with CRC or IBD and healthy controls. Paired cancer and adjacent normal tissues were obtained from CRC patients at the Beijing Friendship Hospital, Capital Medical University.

**TABLE 1:** The demographic data of CRC, IBD and healthy control.

Characteristics	CRC	IBD	Health control
Age, (yr)			
Median (range)	64 (41–84)	49 (16–72)	56 (19–77)
Gender			
Male	60	51	41
Female	37	28	33
Stage			
I	78		
II	11		
III	7		
IV	1		
Total	97	79	74

### 2.2 Cell lines and plasmids

The human CRC cell lines DLD1, HCT116 and RKO were purchased from AnWei‐sci (Shanghai, China) and maintained in DMEM and MEM‐Alpha medium supplemented with 100 U/mL penicillin, 100 mg/mL streptomycin and 10% foetal bovine serum (FBS; BI, Israel) at 37°C in a 5% CO_2_ atmosphere. The pcDNA3.1‐TH1L plasmid and siTH1L (5'‐GGUUGAACUUAUCCGCGUUTT‐3') were designed to regulate TH1L expression. The siCCL20 sequence for human CCL20 was 5'‐CAAGUCAGAAGCAGCAAGCAA‐3'. Plasmids of the NF‐κB reporter and IKKβ ss‐EE were kindly supplied by Zhijie Chang Lab. Other plasmids in this paper were constructed in our lab. The pcDNA3.1‐TH1L plasmid was used to infect cells by lentivirus, and siRNAs were transfected by Lipofectamine 2000 (Invitrogen Life Technologies, Carlsbad, CA) according to the protocol recommended by the manufacturer.

### 2.3 Bioinformatics analysis

We downloaded sample expression profiles and corresponding clinical information data from TCGA database (https://portal.gdc.cancer.gov/, accessed on 5 September 2022) and GEO database (https://www.ncbi.nlm.nih.gov/geo/, accessed on 12 September 2022). By using online analysis tools (https://www.xiantaozi.com), we analysed the expression of TH1L and CCL20 in colorectal cancer samples and normal tissue samples and the correlation between TH1L expression with clinical characteristics and CCL20 expression.

### 2.4 DNA extraction and real‐time PCR

Genomic DNA was extracted from stool samples using a QIAamp Mini Kit (QIAGEN) according to the manufacturer's instructions. The DNA levels of TH1L were assessed by real‐time PCR. Real‐time PCRs were performed using Roche Light Cycler 480 (Rexroth). PCR cycling commenced with one cycle of 95°C for 5 min and 45 cycles of 95°C for 10 s, 60°C for 20 s and 72°C for 20 s.

### 2.5 RNA extraction and cDNA synthesis

Total RNA was extracted with TRIzol (Thermo Scientific, US), and the concentration and purity of RNA were detected using a NanoDrop 2000 (Thermo Scientific, USA). First‐strand cDNA was synthesized from 1 μg of total RNA using a Revert Aid First Strand cDNA Synthesis Kit (BaiMENG, China) according to the manufacturer's protocol. qPCR was performed using Taq SYBR Green Premix (BaiMENG, China) on an ABI Quant Studio6 (Thermo Scientific, USA). GAPDH was used as an endogenous control for normalisation. Real‐time PCR was used to detect the mRNA expression levels of TH1L, CCL20 and the target genes of NF‐κB.

### 2.6 Western blotting

Cells were harvested, and protein was prepared as described previously.^19^ The primary antibodies used were mouse monoclonal anti‐β‐actin (1:1000, Cat# sc‐8432) from Santa Cruz (Dallas Texas, USA), anti‐TH1L (1:1000, Cat# 12265), anti‐IKKβ (1:1000, Cat# 8943), anti‐NF‐κB p65 (1:1000, Cat# 8242S) and anti‐TAK1 (1:1000, Cat# 5206S) from Cell Signalling Technology (Massachusetts, USA), anti‐CCL20 (1:500, Cat# A1756) from ABclonal (Wuhan, China) and anti‐GAPDH (1:1000, Cat# HC301‐01) from Transgen (Beijing, China). Secondary antibodies were used at a 1:5000 dilution and included horseradish peroxidase (HRP)‐conjugated goat anti‐rabbit (OriGene, America) and goat anti‐mouse (OriGene, America) antibodies.

### 2.7 Cell proliferation assay

Cell proliferation was monitored by the IncuCyte ZOOM Live‐Cell Analysis System (Essen, US). Cells were seeded in 96‐well plates at a density of 3000–6000 cells/well and measured for five to 7 days. Each sample had four replicates.

### 2.8 Colony formation assay

For the colony formation assay, the cells were seeded at a density of 500 cells/well in 6‐well plates with 10% FBS‐containing culture medium. The medium was replaced every 3 days. Cells were cultured for 1 or 2 weeks until colonies could be observed. Then, the cells were fixed in 4% paraformaldehyde and stained with 0.1% crystal violet. Colony formation was determined by counting the number of stained colonies. Each experiment was performed at least three times.

### 2.9 Tumour xenografts

Female BALB/c Nude mice that were 5 weeks old were housed in a sterile environment and given bilateral subcutaneous injections of 1 × 10^7^ RKO cells in a saline solution with a volume of 0.1 mL. The tumour volume was calculated using the formula V = 1/2 × larger diameter × (smaller diameter),^2^ which was measured twice a week using a Vernier calliper to determine the tumour's two dimensions. A tumour growth curve was plotted based on the average of the tumour volume (in cubic millimetres). The mice were euthanized for further analysis, and all animal experiments were carried out in compliance with the institution's animal ethics guidelines.

### 2.10 Wound healing assay

The cells were incubated at a density of 5 × 10^4^ cells/well in 96‐well plates and allowed to reach 100% confluence. The cell monolayer was wounded by scratching with a cell scratch apparatus (Essen, US) and then incubated for 1 week. Scratch areas were quantified using the IncuCyte ZOOM Live‐Cell Analysis System (Essen, US). The tests were repeated three times.

### 2.11 Transwell migration assay

Transwell migration assays were performed in 24‐well plates with 8‐μm pore‐sized transwells in accordance with the manufacturer's instructions (Corning, US). The upper chamber was filled with 5 × 10^4^ cells in culture medium without FBS, while the lower chamber coated with collagen I (Corning, US) was filled with culture medium supplemented with 20% FBS. After incubation for 24 h at 37°C, the cells migrating to the lower chamber were stained with haematoxylin and counted by a technician blinded to the experimental settings in four randomly selected microscopic fields of each filter. The tests were repeated three times.

### 2.12 Transcriptome analysis

Total RNA was harvested in cell culture, quantified and qualified by 1% agarose gels, the NanoPhotometer® spectrophotometer (IMPLEN, CA, USA) and the RNA Nano 6000 Assay Kit of the Bioanalyzer 2100 system (Agilent Technologies, CA, USA). A total amount of 1 μg RNA per sample was used as input material for the RNA sample preparations. Sequencing libraries were generated using the NEBNext® UltraTM RNA Library Prep Kit for Illumina® (NEB, USA) following the manufacturer's recommendations, and index codes were added to attribute sequences to each sample. Data analysis mainly focused on differential expression analysis and functional enrichment analysis.

### 2.13 ELISA assay

Cell supernatants were collected and centrifuged for 10 min at 1000 rpm and 4°C. The cell‐free supernatants were analysed by a CCL20/MIP 3‐alpha sandwich ELISA Kit (ABclonal, China). CCL20 concentrations were calculated based on a standard curve that was obtained by using defined CCL20 concentrations (0, 7.8, 15.6, 31.25, 62.5, 125 and 250 pg/mL).

### 2.14 Luciferase reporter assay

HEK‐293 T cells were inoculated into a 24‐well plate, and when the confluence reached 70%, the plasmids were cotransfected with the VigoFect Transfection Reagent (Vigorous Biotechnology Beijing Company, China). After 24 h, the cells were lysed and measured by a Vigorous luciferase assay kit (Vigorous Biotechnology Beijing Company, China), and the luciferase activity was obtained by an Infinite F200 pro (Tecan, Switzerland). Luciferase activity was normalised to Renilla for each sample. Each experiment was repeated three times.

### 2.15 Statistics

A chi‐squared test was used for categorical variables, and Student's *t* test was used for continuous variables. Differences were considered statistically significant at *p* < 0.05. All statistical analyses were performed using SPSS version 20.0 software.

## 3 RESULTS

### 3.1 Stool and tissue TH1L expression was increased in CRC

**FIGURE 1:**
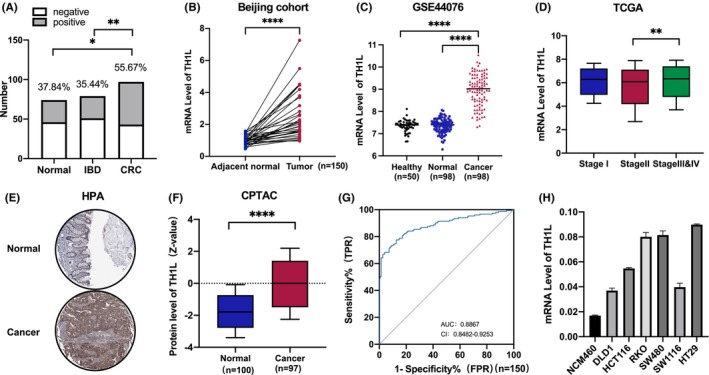
TH1L expression was increased in colorectal cancer. (A) The DNA level of TH1L in CRC patients (*n* = 97), IBD patients (*n* = 79) and normal controls (*n* = 74). (B–C) The mRNA levels of TH1L were analysed in the tumours compared with their paired adjacent normal tissues from the Beijing cohort (*n* = 150) (shown in B) and GSE44076 (shown in C). (D) Box plot of TH1L expression in tumours with different histological grades is shown from the TCGA database. (E) Representative immunohistochemistry image of TH1L protein expression in CRC tissues (Cancer) and their adjacent tissues (Normal) from the HPA database. (F) The protein levels of TH1L were analysed in tumour tissues compared with normal tissues from the CPTAC database. (G) Receiver operating characteristic (ROC) curves for detecting CRC patients in the Beijing cohort (*n* = 150). (H) The expression of TH1L was measured by qRT–PCR in normal and CRC cells. *, *p* < 0.05; **, *p* < 0.01; ***, *p* < 0.001; ****, *p* < 0.0001.

To explore the status of *Th1l* in colorectal cancer, we first investigated the amplification of the *Th1l* gene in stool samples from patients with CRC (*n* = 97) or IBD (*n* = 79) and healthy controls (*n* = 74). The results showed that *Th1l* DNA was detected in 54 CRC patients, 28 IBD patients and 28 healthy controls. Compared to that in IBD patients and healthy controls, the rate of *Th1l* DNA detection was significantly higher in CRC patients (Figure 1A). Furthermore, we examined the mRNA expression of TH1L in 150 paired human samples of colorectal cancer and adjacent normal tissue (Beijing cohort). The mRNA level of TH1L was obviously higher in the cancer tissues than in the adjacent normal tissues (Figure 1B), which was confirmed in GEO databases from CRC patients (Figure 1C). Bioinformatic analyses from TCGA revealed that TH1L mRNA expression was significantly increased in the progression to later clinical stages of CRC (Figure 1D). Consistent with these data, immunohistochemical analyses showed that the expression of TH1L protein was higher in CRC samples than in normal tissues using the HPA and CPTAC databases (Figure 1E,F). The data demonstrated that TH1L was significantly upregulated in CRC. To further validate this, receiver operating characteristic (ROC) curve analysis was also performed (Figure 1G), which revealed that the mRNA level of TH1L was sufficient to differentiate CRC tissues from normal tissues. Additionally, the TH1L mRNA level was examined in six CRC cell lines and the colorectal epithelial cell line NCM460. The expression of TH1L was higher in all CRC cell lines than in normal colorectal epithelial cells (Figure 1H). In conclusion, these results suggested that TH1L is upregulated in CRC cells, tissues and faeces from CRC patients.

### 3.2 TH1L enhanced the proliferation of CRC cells

**FIGURE 2:**
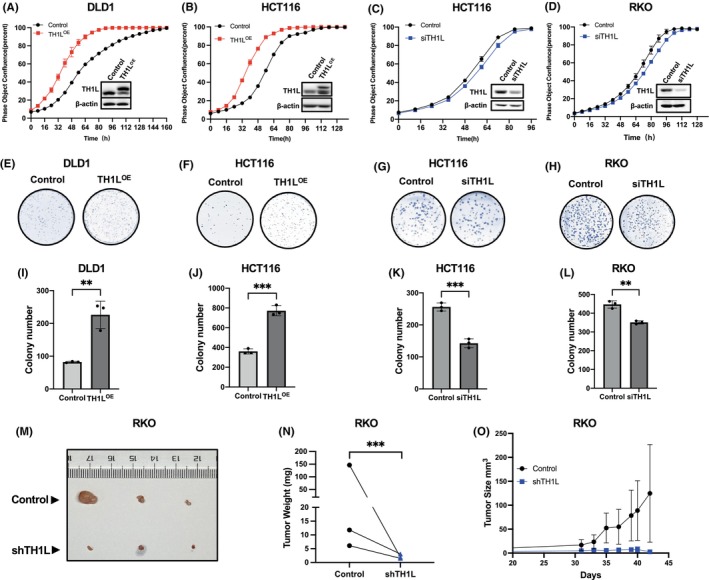
TH1L promotes colorectal cancer cell growth. (A–D) The cell growth curves of TH1L‐overexpressing DLD1 and HCT116 cells and TH1L‐knockdown HCT116 and RKO cells were determined through IncuCyte. The expression of TH1L was confirmed by western blotting. (E–L) The colony formation capacity of TH1L‐overexpressing DLD1 and HCT116 cells and TH1L‐knockdown HCT116 and RKO cells was determined by counting the colonies. Photographs of colonies are shown in (E–H). Quantitative analyses are shown in (I–L). All experiments were repeated three times. *, *p* < 0.05; **, *p* < 0.01; ***, *p* < 0.001; ****, *p* < 0.0001. (M–O) Knocking down TH1L suppresses xenograft tumour growth. Nude mice were injected on the left side with control cells and the right side with TH1L‐depletion (shTH1L) RKO cells using 1 × 10^7^ cells. The xenograft tumours were measured over times and dissected at day 42. The final tumour volumes (M) and quantification of tumour weight (N) and tumour size (O) were shown, respectively (*n* = 3).

To investigate whether the expression level of TH1L was associated with the proliferation of CRC, we used three different cell lines: DLD1 with a low expression level of TH1L, RKO with a high level of TH1L and HCT116 with a medium level of TH1L (Figure 1H). We overexpressed TH1L in DLD1 and HCT116 cells and knocked down endogenous TH1L in HCT116 and RKO cells. Western blotting assays confirmed the successful construction of the CRC cell lines (Figure 2A–D, bottom right). A cell proliferation assay and CCK8 assay indicated that overexpression of TH1L in DLD1 and HCT116 cells promoted (Figure 2A,B and Figure S1A,B), while knockdown of TH1L in HCT116 and RKO cells inhibited (Figure 2C,D and Figure S1C,D), cell proliferation. Moreover, the colony formation assay indicated that TH1L overexpression enhanced the ability of CRC cells to form colonies (Figure 2E,F,I,J), while silencing TH1L significantly inhibited colony formation (Figure 2G,H,K,L). Furthermore, the effect of TH1L on tumour formation was evaluated via tumour xenograft experiment in vivo. The results showed that knocking down TH1L cells formed smaller tumours (Figure 2M,N). Tumour growth curves illustrated slower growth by TH1L‐knockdown cells (Figure 2O). Taken together, these data suggested that TH1L remarkably promotes cell growth in CRC cells.

### 3.3 TH1L accelerated the migration of CRC cells

**FIGURE 3:**
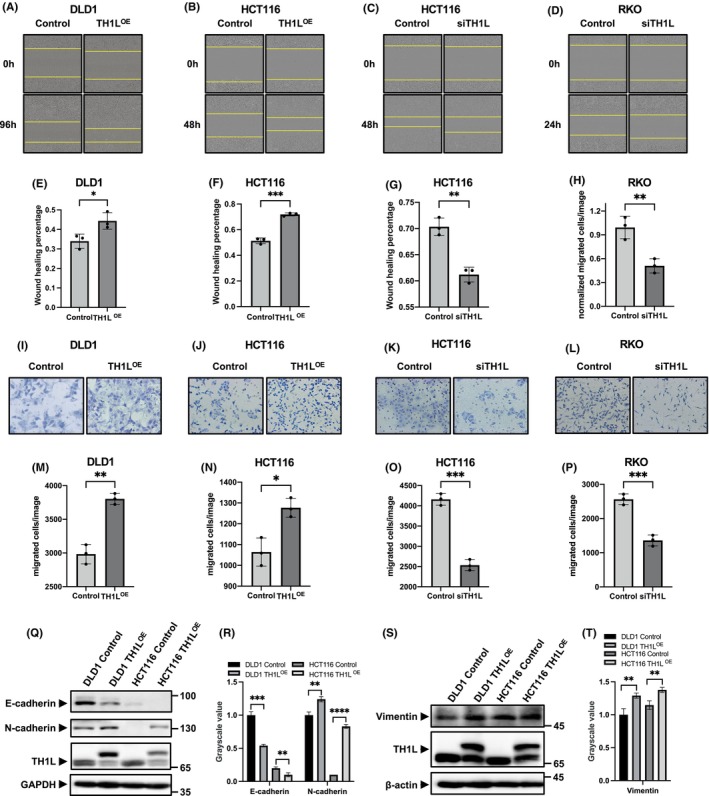
TH1L promotes colorectal cancer cell migration. (A–H) The capacity of cell migration was assessed by wound healing assay. Photographs of representative wound healing are shown in (A–D). Quantitative analyses of the wound healing percentage are shown in (E–H). (I–P) Cell invasion was determined by transwell assay. Photographs of representative cancer cells that migrated to the bottom side of the membrane are shown in (I–L). (M–P) display the number of invaded cells. (Q) The protein expression of E‐cadherin and N‐cadherin was assessed by Western blotting when TH1L was overexpressed. (R) Bar chart of the banding grayscale value of (Q). (S) The Vimentin protein level was assessed by Western blotting when TH1L was overexpressed. (T) Bar chart of the banding grayscale value of (S). *, *p* < 0.05; **, *p* < 0.01; ***, *p* < 0.001; ****, *p* < 0.0001.

To assess the migratory capacity of cells, we conducted a wound‐healing assay. As shown in Figure 3, overexpression of TH1L significantly promoted the migration of DLD1 cells and HCT116 cells (Figure 3A,B), while the opposite results were observed in HCT116 cells and RKO cells with TH1L knockdown (Figure 3C,D). Quantitative analyses confirmed a considerable increase in wound closure in the TH1L‐overexpressing cells compared to the control cells, thereby indicating that the migration rate of the TH1L‐overexpressing cells was significantly higher than that of the control cells (Figure 3E,F). As expected, a decrease in the healing rate was observed in the TH1L knockdown group (Figure 3G,H).

To further validate the impact of TH1L on cell migration, we performed a transwell assay and found that overexpression of TH1L in DLD1 and HCT116 cells led to accelerated migration of the cells (Figure 3I,J,M,N). In contrast, silencing TH1L drastically repressed CRC cell migration (Figure 3K,L,O,P). Subsequently, the expression of EMT‐related proteins was also detected by western blotting. The results revealed that when TH1L is overexpressed, there is a downregulation in the protein levels of E‐cadherin and an upregulation in the protein levels of both N‐cadherin and Vimentin (Figure 3Q–T). Collectively, these data demonstrated that TH1L enhanced the migration ability of CRC cells.

### 3.4 CCL20 acted as a target of TH1L

To further explore the mechanisms underlying its oncogenic activity, we conducted transcriptome analysis of TH1L‐overexpressing DLD1 cells and HCT116 cells. We filtered TH1L‐coexpressed genes from the results report and then performed GO, KEGG and Reactome pathway analysis on those genes, which were positively correlated with TH1L and enriched in processes such as DNA replication, mitotic spindle organisation, cell cycle, etc. (Figure S2A,B). These findings suggested that TH1L was associated with a range of cancer‐associated pathways.

**FIGURE 4:**
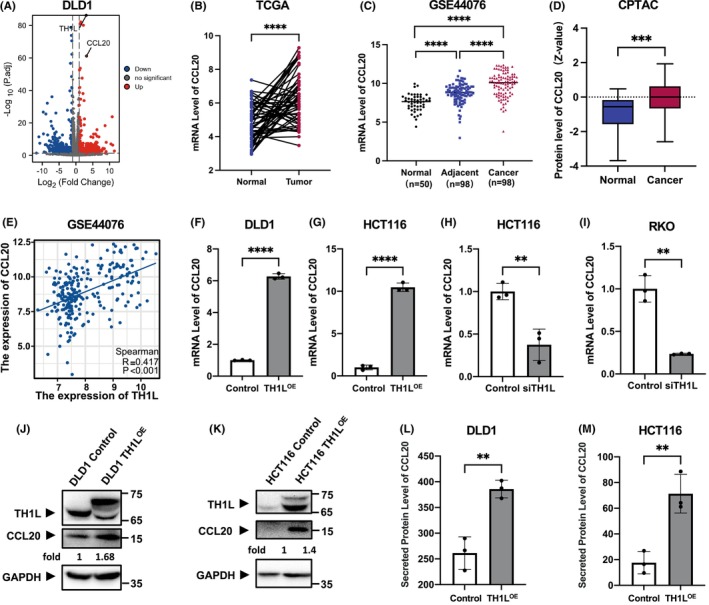
TH1L regulates CCL20 expression in CRC cells. (A) Volcano maps displaying fold changes in gene expression in TH1L‐overexpressing DLD1 cells compared with negative control cells. (B, C) The mRNA levels of CCL20 were analysed in the tumours compared with their paired adjacent normal tissues from the TCGA database (shown in B) and GSE44076 (shown in C). (D) CCL20 protein levels were analysed in tumour tissues compared with normal tissues from the CPTAC database. (E) Analysis of the correlation between TH1L and CCL20 expression levels in CRC tissues in GSE44076. The expression of CCL20 was determined by qRT–PCR (F–I), Western blot (J, K) and ELISA (L, M) after TH1L was overexpressed and knocked down in CRC cells. All experiments were repeated three times. *, *p* < 0.05; **, *p* < 0.01; ***, *p* < 0.001; ****, *p* < 0.0001.

To explore its specific mechanism, we further analysed the genes with large changes (Log2Fold Change >0.5). Among the genes upregulated by TH1L, C‐C motif chemokine ligand 20 (CCL20) was significantly overexpressed (Figure 4A and Figure S2C). Therefore, CCL20 was selected for further experimental validation. At the mRNA and protein levels, the expression of CCL20 in cancer tissues was higher in CRC samples than in normal tissues using the TCGA, GEO and CPTAC databases (Figure 4B–D). A positive correlation between TH1L and CCL20 mRNA expression was confirmed in GEO databases (Figure 4E). Real‐time PCR showed that CCL20 expression was positively correlated with the level of TH1L (Figure 4F–I). Subsequently, we assessed the protein level of CCL20 using western blotting and ELISA (Figure 4J–M). The elevation of CCL20 protein expression was validated in TH1L‐expressing DLD1 and HCT116 cells. These data verified that CCL20 was a downstream target of TH1L in CRC cells.

### 3.5 TH1L enhanced colorectal cancer proliferation and migration by activating the NF‐κB signalling pathway

**FIGURE 5:**
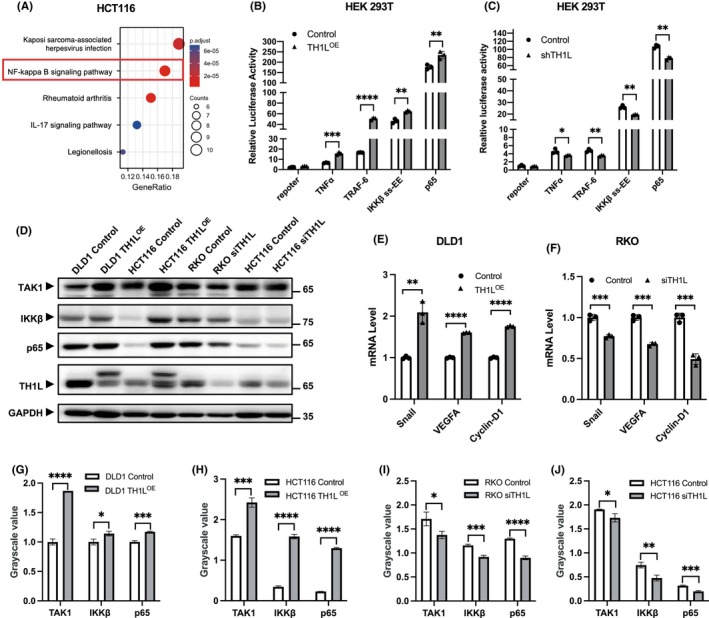
TH1L promotes the activation of the NF‐κB signalling pathway. (A) Results of hallmark signalling pathway enrichment analysis. The size of the dots represents the number of genes in different hallmark pathways, and the colours of the dots indicate the change in the adjusted *p* value. (B, C) The activity of the NF‐κB signalling pathway in HEK 293 T cells was confirmed by luciferase reporter assay after TH1L overexpression (shown in B) or knockdown (shown in C). (D) The protein expression of TAK1, IKKβ and p65 was detected in TH1L‐overexpressing DLD1 and HCT116 cells and TH1L‐knockdown RKO and HCT116 cells by western blotting. GAPDH was used as the loading control. (E, F) The mRNA levels of NF‐κB signalling pathway target genes were assessed by qRT–PCR when TH1L was overexpressed and knocked down. (G–J) Bar charts of the banding grayscale value of (D). All experiments were repeated three times. *, *p* < 0.05; **, *p* < 0.01; ***, *p* < 0.001; ****, *p* < 0.0001.

Numerous studies have indicated that CCL20 contributes to tumour cell invasion and metastasis through the activation of the NF‐κB signalling pathway.^16^ Meanwhile, our enrichment analysis of RNA‐seq also revealed that the significantly upregulated genes were enriched in the NF‐κB signalling pathway (Figure 5A).

To investigate whether TH1L promotes the NF‐κB signalling pathway, luciferase assays were performed in HEK293T cells. The results showed that after TH1L overexpression, the relative luciferase activity was elevated (Figure 5B). In addition, relative luciferase activity was decreased upon transfection with the shTH1L plasmid (Figure 5C). The protein levels of TAK1, IKKβ and p65 were observed to be increased in DLD1 and HCT116 cells upon overexpression of TH1L. Conversely, knockdown of TH1L in RKO cells decreased the amount of these proteins (Figure 5D,G–J). Moreover, overexpression of TH1L resulted in an increase in the target genes of the NF‐κB signalling pathway, including Snail, VEGFA and CyclinD1, while knockdown of TH1L showed the opposite effect (Figure 5E,F). Taken together, these results indicated that the NF‐κB pathway is activated in the process of TH1L‐induced proliferation and migration of CRC cells.

### 3.6 TH1L increased colorectal cancer proliferation and migration by upregulating CCL20 expression

**FIGURE 6:**
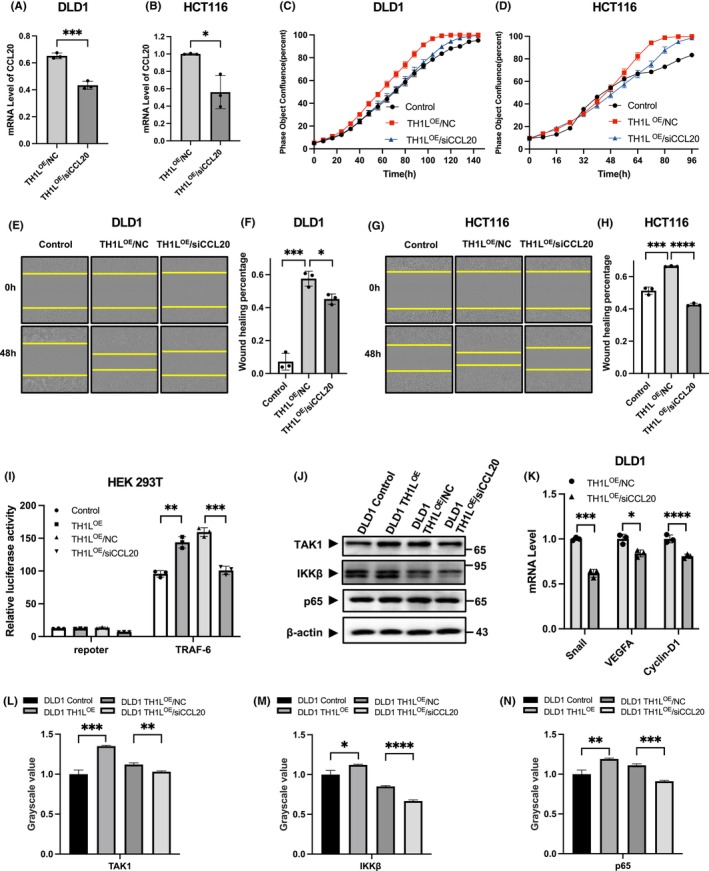
TH1L promotes tumour cell proliferation and migration by upregulating CCL20. (A, B) The expression of CCL20 was confirmed by RT–qPCR in TH1L‐overexpressing DLD1 and HCT116 cells. (C, D) The cell proliferation curves were measured in TH1L‐overexpressing DLD1 and HCT116 cells with CCL20 knockdown by IncuCyte. (E–H) The capacity of cell migration assessed in TH1L‐overexpressing DLD1 and HCT116 cells with CCL20 knockdown by wound healing tests. (I) NF‐κB signalling pathway activity was confirmed by luciferase reporter assay after knockdown of CCL20 in TH1L‐overexpressing HEK293T cells. (J) The protein expression of TAK1, IKKβ and p65 was detected in TH1L‐overexpressing DLD1 cells with CCL20 knockdown by western blotting. (K) The mRNA levels of Snail, VEGFA and CyclinD1 were assessed in TH1L‐overexpressing DLD1 cells with CCL20 knockdown by qRT–PCR. (L–N) Bar charts of the banding grayscale value of (J). All experiments were repeated three times. *, *p* < 0.05; **, *p* < 0.01; ***, *p* < 0.001; ****, *p* < 0.0001.

Since CCL20 is supposed to be a target gene of TH1L, we then performed functional recovery experiments to determine whether TH1L exerts its functions by modulating CCL20 expression and the NF‐κB signalling pathway in CRC. HCT116 and DLD1 cells overexpressing TH1L were transfected with siCCL20 (Figure 6A,B), and then the cell proliferation and migration abilities were measured.

The data indicated that the enhanced proliferation and migration could be reversed when CCL20 was knocked down (Figure 6C–H). To validate our hypothesis, we further detected the NF‐κB signalling pathway by luciferase assay and Western blotting. The results showed that the relative luciferase activity and the protein levels of TAK1, IKKβ and p65 were affected by TH1L expression, and this effect was impaired by the knockdown of CCL20 (Figure 6I,J). The expression of NF‐κB signalling pathway target genes also changed in the same trend (Figure 6K,L–N). These data confirmed that TH1L promoted CRC proliferation and migration by regulating CCL20 expression and the NF‐κB signalling pathway.

## 4 DISCUSSION

This study elucidates that TH1L can play a pro‐oncogenic role in CRC. We found not only that the expression of TH1L was significantly high in CRC tissues but also that stool samples from CRC patients had a higher rate of TH1L DNA positivity than those from patients with IBD and healthy controls. Meanwhile, we identified a novel mechanism by which TH1L induces the activation of NF‐κB through CCL20 upregulation, leading to enhanced proliferation and migration in CRC cells. In summary, TH1L may be a potential biomarker and therapeutic target for CRC.

Our cellular studies and clinical observations consistently support the protumor role of TH1L in CRC. However, previous studies have shown that upregulation of TH1L suppresses the growth and migration of human breast cancer cells. Furthermore, the knockdown of TH1L resulted in downregulation of p21 and upregulation of p‐ERK and β‐catenin expression in breast cancer cells.^12^ We then determined the levels of these genes in CRC cells, which exhibited a different trend (Figure S2D). Thus, TH1L in different cancer types appears to drive distinct downstream gene expression patterns. Moreover, amplification and overexpression of genes on chromosome 20q are common in CRC^20^, ^21^, ^22^ and are highly correlated with unfavourable prognoses in CRC.^23^, ^24^, ^25^ Intriguingly, TH1L is located at 20q13, which is consistent with the results reported by Song. et al.^14^ Therefore, TH1L seems to have opposing roles in different tumour types.

In our potential mechanistic study of TH1L, we discovered that CCL20 is a key target of TH1L, which promotes intestinal tumorigenesis and CRC metastatic progression.^15^, ^26^, ^27^, ^28^ By conducting RNA sequencing analysis, we found that CCL20 displayed the highest level of expression among the upregulated genes in DLD1 cells. Further testing showed a significantly positive correlation between TH1L expression and CCL20. Knocking down CCL20 reversed TH1L‐promoted cell proliferation and migration. However, pathway enrichment analysis of differentially expressed genes in TH1L‐overexpressing CRC cells showed enrichment of genes involved in DNA replication, the cell cycle and amplified inflammatory factors (Figure S2A,B). Although CCL20 is an important target for TH1L to promote CRC cell proliferation, it is not the only downstream target. Therefore, further studies are needed to investigate other potential downstream targets and their underlying mechanisms.

The NF‐κB signalling pathway plays a crucial role in various stages of colon cancer development, including epithelial‐mesenchymal transformation, invasion, metastasis, cancer stem cell formation, cell metabolism and chemotherapy resistance.^29^, ^30^, ^31^ Notably, activation of the NF‐κB signalling pathway is responsible for the regulation of CCL20‐mediated proliferation, invasion and metastasis of many types of cancer cells.^16^, ^27^, ^32^ Our RNA‐sequencing‐based enrichment analysis revealed that upregulated genes were significantly enriched in the NF‐κB signalling pathway. This was further supported by the elevated protein levels of NF‐κB pathway members and the transcription of NF‐κB target genes. The NF‐κB signalling pathway and CCL20 are both associated with immunity.^26^, ^31^, ^33^, ^34^ Additionally, our RNA‐seq data showed that TH1L alters the expression of multiple secreted cytokines and chemokines in CRC. Therefore, these results suggest that TH1L may contribute to the regulation of the proportion of various immune cells in the tumour microenvironment. However, the exact mechanisms underlying this observation remain to be further elucidated.

Stool‐based TH1L DNA testing identified 55.67% of patients with CRC in the Beijing cohort. Additionally, detection rates were significantly higher among CRC patients than among those with inflammatory bowel disease (IBD). These findings suggest that TH1L may serve as a predictive biomarker in clinical settings. There have been studies indicating that stool DNA testing, which is noninvasive, cost‐effective and more sensitive than faecal occult blood testing, should be considered as a screening approach for colorectal cancer.^35^, ^36^, ^37^ Further studies are required to address the specific relationship between stool DNA level and stages of cancer in the clinical setting and to assess whether it can be used for early detection.

## AUTHOR CONTRIBUTIONS

**Yu Shang:** Conceptualization (equal); methodology (equal); project administration (equal); resources (equal); supervision (equal); writing – original draft (equal); writing – review and editing (equal). **Haiyun Shi:** Conceptualization (equal); methodology (equal); project administration (equal); resources (equal); supervision (equal); writing – review and editing (equal). **Shaochang Wang:** Investigation (equal); validation (equal); visualization (equal); writing – original draft (equal). **Yujing Sun:** Investigation (equal); visualization (equal). **Chunya Li:** Validation (equal). **Yueyang Chong:** Validation (equal). **Meihong Ai:** Validation (equal). **Yanxia Wang:** Validation (equal).

## CONFLICT OF INTEREST STATEMENT

The authors declare no conflict of interest.

## Supporting information


Figures S1–S2.

## Data Availability

The data and materials that support the findings of this study are available from the corresponding author upon reasonable request.
