# Supplementary material for: TH1L involvement in colorectal cancer pathogenesis by regulation of CCL20 through the NF‐κB signalling pathway
Source: J Cell Mol Med. 2024 May 29;28(10):e18391. doi: 10.1111/jcmm.18391 (PMC11135906; doi:10.1111/jcmm.18391)
Supplement: Supplementary file 1 — Figures S1–S2. [file JCMM-28-e18391-s001.docx]

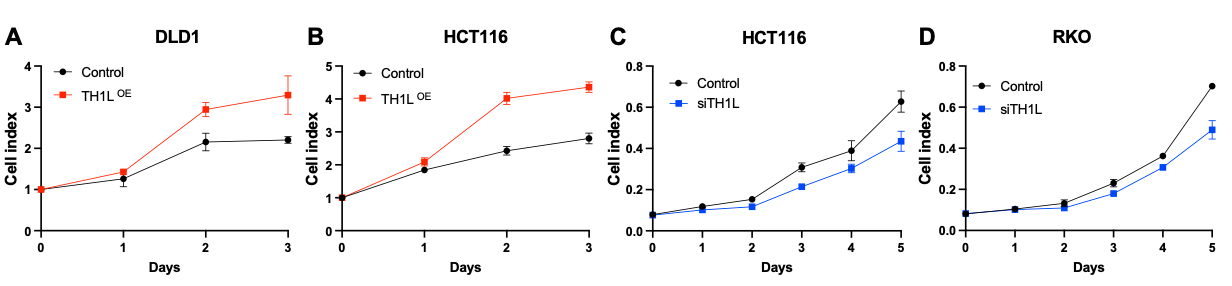


**Sup Figure S1. (A-B)** The proliferation of negative control (Control) or TH1Loverexpressed HCT116 and RKO cells (TH1L^OE^) were tested by CCK8 regent (n = 3). **(C-D)** The proliferation abilities of HCT116 and RKO cells transfected with siTH1L (siTH1L) or NC (Control) were determined using CCK8 assay (n = 3).


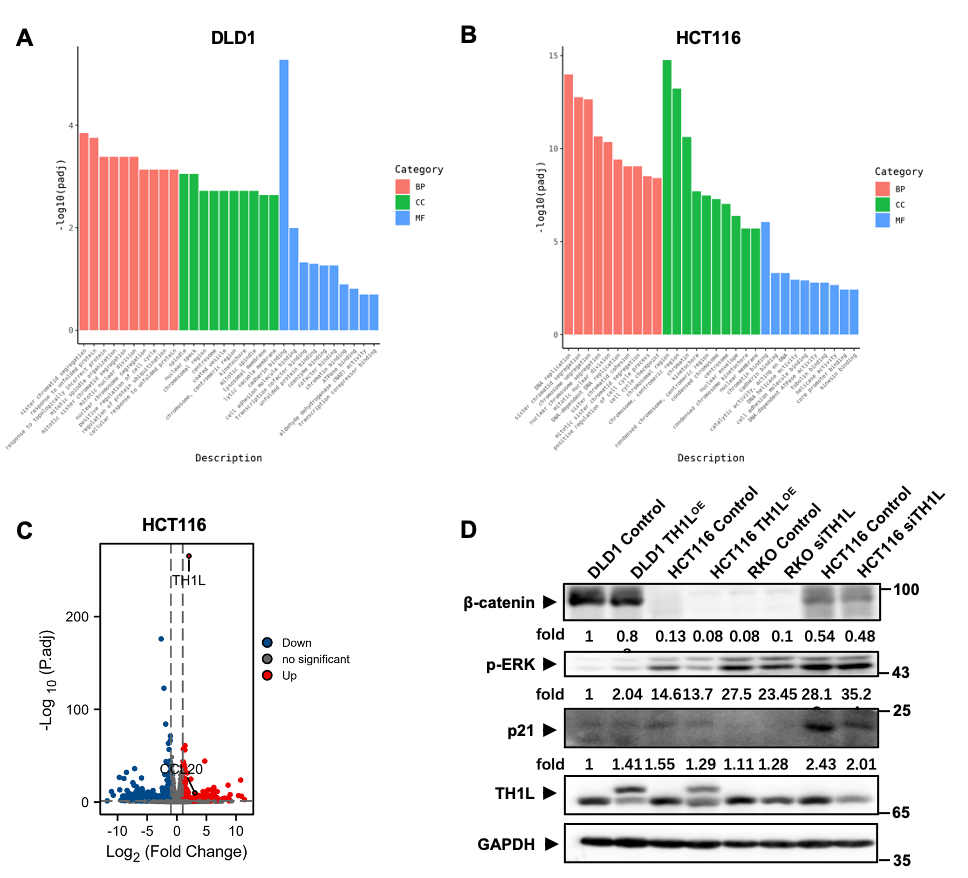


**Sup Figure S2. (A-B)** GO analysis of all changed genes in DLD1(A) and HCT116 (B) TH1L-overexpressed cells compared with control cells. **(C)** Scatter plot displayed fold changes of gene expression in TH1L-overexpressed HCT116 cells compared with negative control cells. **(D)** Western blot analyses of expression of β-catenin, p-ERK and p21 in control and TH1L-overexpressing DLD1 and HCT116 cells or in RKO and HCT116 cells transfected with NC or TH1L siRNA.
